# Roles of Resveratrol in Improving the Quality of Postovulatory Aging Oocytes In Vitro

**DOI:** 10.3390/cells8101132

**Published:** 2019-09-23

**Authors:** Yan-Li Sun, Shou-Bin Tang, Wei Shen, Shen Yin, Qing-Yuan Sun

**Affiliations:** 1 College of Animal Science and Technology, Qingdao Agricultural University, Qingdao 266109, China; 2 College of Life Sciences, Institute of Reproductive Science, Key Laboratory of Animal Reproduction and Germplasm Enhancement in Universities of Shandong, Qingdao Agricultural University, Qingdao 266109, China; 3 State Key Laboratory of Stem Cell and Reproductive Biology, Institute of Zoology, Chinese Academy of Sciences, Beijing 100101, China

**Keywords:** oocyte, postovulatory aging, resveratrol, Juno

## Abstract

After ovulation, mammalian oocytes will undergo a time-dependent process of aging if they are not fertilized. This postovulatory aging (POA) seriously affects the oocyte quality and then impairs the subsequent fertilization and early embryo development, which should be avoided especially in assisted reproductive technology (ART). Resveratrol is an antioxidant substance that can scavenge free radicals and is effective in improving ovary functions. Here, mouse oocytes were used to investigate the effects and mechanisms of resveratrol on POA oocytes in vitro. With 1.0 µM resveratrol treatment during aging process, the rates of fertilization and blastocyst in POA oocytes increased significantly compared with those in the POA group. Resveratrol can reduce the loss of sperm binding sites by stabilizing Juno. Resveratrol can maintain the normal morphology of spindle and mitochondrion distribution and alleviate the levels of ROS and early apoptosis. Additionally, resveratrol can reduce the changes of H3K9me2. Therefore, resveratrol can significantly improve the quality of POA oocytes in vitro to enhance the rates of fertilization and blastocyst, which may be very helpful during the ART process.

## 1. Introduction

In the whole modern world, the delayed childbearing or reproductive diseases considerably contribute to the increase in the number of people who require assisted reproductive technology (ART) [1,2]. In this process, the metaphase of second meiosis (MII) stage oocytes need to be cultured for a period of time in vitro, during which they will present the phenomenon of “oocyte overripeness” [3]. This means if the mature oocytes were not fertilized or activated in time, they will progressively undergo a time-dependent process of aging, according named postovulatory aging (POA) [4]. This inevitably impairs the quality of oocytes. Obviously, POA in vitro has adverse effects on ART.

Previous studies have shown that POA oocytes in vitro are always accompanied by diverse biochemical changes. For example, POA leads to the incompact cumulus expansion and fragmentation [5], increases the level of reactive oxygen species (ROS) [6,7,8], decreases the expression of anti-apoptotic factor BCL-2 [9] and activates maturation promoting factor (MPF) in oocytes [10,11]. Also, nuclear organization is affected in stress and aging oocytes [12]. All these changes can seriously impair the oocyte quality and subsequent embryo development [13,14], which inevitably affects the ART success. Therefore, it is very urgent and important to search some effective ways to reduce the damage caused by POA in vitro.

Resveratrol (3,5,4′-trihydroxystilbene) is a well-known polyphenolic compound and a secondary metabolite from peanuts, berries, grapes, and red wine (the most important ingredient in wine). It is usually used as a nutritional supplement and medicinal ingredient [15]. Resveratrol is also well recognized for antioxidant and anti-aging substance because it scavenges free radicals. The previous studies have reported that resveratrol is effective in improving ovary functions [16]. Resveratrol is an anti-apoptotic factor in organisms through several different pathways including interleukin family [17], the signal of p53 [18], and ROS-dependent pathways [19]. It has been shown that resveratrol can activate the Sirt3 to promote the function of mitochondria by modulating acetylation of mitochondrial transcription factor (TFAM) [20], affect methylglyoxal adduct accumulation and migration/invasion in anaplastic thyroid cancer (ATC) cells in vitro [21], and enhance the longevity of several species [22]. Additionally, resveratrol is reported to promote the development of embryo from vitrified mouse oocytes after in vitro fertilization (IVF) [23]. Therefore, it is reasonable to utilize resveratrol for improving the quality of POA oocytes in vitro. However, no studies have been reported about the effects of resveratrol on POA oocytes in vitro, which simulate ART.

In this study, the mouse oocytes were cultured with resveratrol during POA in vitro and then examined by several assays. The results show that resveratrol can significantly enhance the rates of fertilization and blastocyst in vitro by improving the sperm binding ability via Juno stabilization, the correct spindle assembly, normal mitochondrial functions and epigenetic modification changes. Therefore, resveratrol can effectively improve the quality of oocytes during POA in vitro and enhance the rates of fertilization and blastocyst. This will be very helpful in ART.

## 2. Materials and Methods

### 2.1. Animals and Feeding

All the procedures were approved by the Animal Research Committee of Qingdao Agricultural University. Four-week-old ICR (Institute of Cancer Research, Philadelphia, USA) female mice and eight-week-old ICR male mice were raised in a temperature-controlled room with normal dark-light cycles and fed with a regular diet. During the ovary and epididymis collection, pain relief was considered, and the mice were humanely treated.

### 2.2. Oocyte Collection and Blastocyst Culture

In order to obtain MII oocytes, 4–6 weeks ICR female mice were given 10 IU PMSG (Ningbo Hormone Product Co., Ltd., P.R. China), and after 48 h given 10 IU hCG (Ningbo Hormone Product Co.). Oviductal ampullae was taken out at 13 h after hCG injection and broken by syringe to release the cumulus oocyte complexes (COCs). Cumulus cells of COCs were removed in M2 medium supplement with 0.1% hyaluronidase (Sigma-Aldrich, St. Louis, MO, USA). Then oocytes with the first polar body were cultured in KSOM medium (EMD Millipore Corp, Billerica, MA, USA) in a 5% CO_2_ atmosphere at 37 °C for IVF and early embryo development. Pronuclei were observed at 8 h after IVF. Fertilized oocytes developed to 2-cell stage at 12 h and blastocyst stage at 4.5 d after IVF.

### 2.3. In Vitro Fertilization

Cauda epididymides were scratched in a dish of human tubal fluid (HTF) medium to release sperms. After capacitation in HTF for 1 h with 5% CO_2_ at 37 °C, 1 × 10^6^/mL sperms in supernatant were added to droplets of KSOM medium containing the oocytes for 4–6 h. Only these oocytes with two pronuclei were considered as successful fertilization.

### 2.4. POA In Vitro and Resveratrol Treatment

MII oocytes were aging for 0 h, 6 h, and 12 h in vitro in 35 µL droplets of KSOM under mineral oil at 37 °C with 5% CO_2_. According to the rate of blastocyst after IVF, we chose the POA 12 h as the time point to perform resveratrol experiments. Resveratrol (J&K, Beijing, China) was dissolved in DMSO at a stock concentration of 100 mM and diluted as the final concentration of 0, 0.1, 1, or 10 µM. The oocytes were aged for 12 h in vitro with resveratrol at 0, 0.1, 1, and 10 µM, and then all the procedures were same to the fresh MII oocytes as described above. For all treatments, the biggest concentration of DMSO is less than 0.01% in the culture medium and the results showed no significant difference compared with the control group [24]. All the experimental conditions were same.

### 2.5. Sperm Binding Assay

The capacitated sperms (1 × 10^6^/mL) were added to the control, POA 12 h and resveratrol-treated POA 12 h (POA + Res) oocytes to incubate additional 1 h. Then the samples were fixed in 4% PFA for 30 min and stained with DAPI for 15 min. Sperms bound to the oocyte were quantified from z projections acquired by a laser scanning confocal microscope (Leica TCS SP5, Mannheim, Germany). DAPI-positive point indicates one sperm bound to the oocyte successfully. Each group contained 25–30 oocytes [25].

### 2.6. Immunofluorescent Staining

Oocytes were fixed in 4% PFA for 30 min and permeabilized in phosphate-buffered saline (PBS) supplement with 0.5% Triton X-100 for 20 min at room temperature. After blocking in PBS with 0.1% bovine serum albumin (BSA) for 1 h, oocytes were incubated with primary antibody at 4 °C overnight. After washing 3–5 times in PBS containing 1% Tween 20 and 0.01% Triton-X 100, oocytes were incubated with an appropriate secondary antibody at room temperature for 2 h. Then, after washing 3–5 times, oocytes were stained with DAPI for 15 min. Finally, oocytes were transferred to glass slides with a DABCO drop and observed by using a laser scanning confocal microscope. The primary antibodies included rat monoclonal anti-mouse Folr4-FITC antibody (Juno, 1:100, BioLegend, San Diego, CA, USA), mouse monoclonal anti-α-tubulin FITC antibody (1:500, Santa Cruz Biotechnology, Dallas, TX, USA), rabbit polyclonal anti-H3K9me2 antibody (1:100, Bioworld Technology Inc., St. Louis Park, MN, USA). At least 3 replicates were performed and oocytes not less than 20 were observed for each group [26].

### 2.7. Detection of ROS Level

Oocytes were processed by using the oxidation-sensitive fluorescent probe at 37 °C for 30 min according to the Reactive Oxygen Species Assay Kit (Beyotime Institute of Biotechnology, Shanghai, China). To detect the fluorescent intensity of ROS, the confocal system was used with the same scanning settings [27].

### 2.8. Annexin-V Staining

Apoptosis analysis was evaluated by using the Annexin V-FITC Apoptosis Kit (Beyotime Institute of Biotechnology). Oocytes were stained with 195 µL binding buffer containing 5 µL Annexin-V FITC for 30 min in the dark. After washing 3 times, oocytes were observed under a laser scanning confocal microscope to detect the fluorescent signals at the membrane. The fluorescent signal on the membrane was considered as the symbol of early apoptosis [28].

### 2.9. Mitochondrion Distribution

To observe the mitochondrion distribution, oocytes were stained with MitoTracker Deep Red (Invitrogen, Carlsbad, CA, USA). After being put in pre-warmed staining solution for 30 min at 37 °C, oocytes were stained with DAPI for 15 min. At least 25 oocytes for each group were observed under a laser scanning confocal microscope. The mitochondrion distribution is counted according to previously described [29].

### 2.10. Fluorescence Intensity Analysis

All pictures were captured by using the same scanning settings. Per unit area within the region of interest (ROI) of the average fluorescence intensity was examined by image J software (v.1.47, National Institutes of Health, Bethesda, MD, USA).

### 2.11. Statistical Analysis

Each group contained 25–30 oocytes. All data from at least three replicated experiments were expressed as mean ± SEM and analyzed with Graph Pad Prism software (v.6.01) by one-way of variance (ANOVA) analysis. *P* value less than 0.05 was considered as significant difference.

## 3. Results

### 3.1. Effects of POA In Vitro on the Rates of IVF and Early Embryo Development

**Figure 1:**
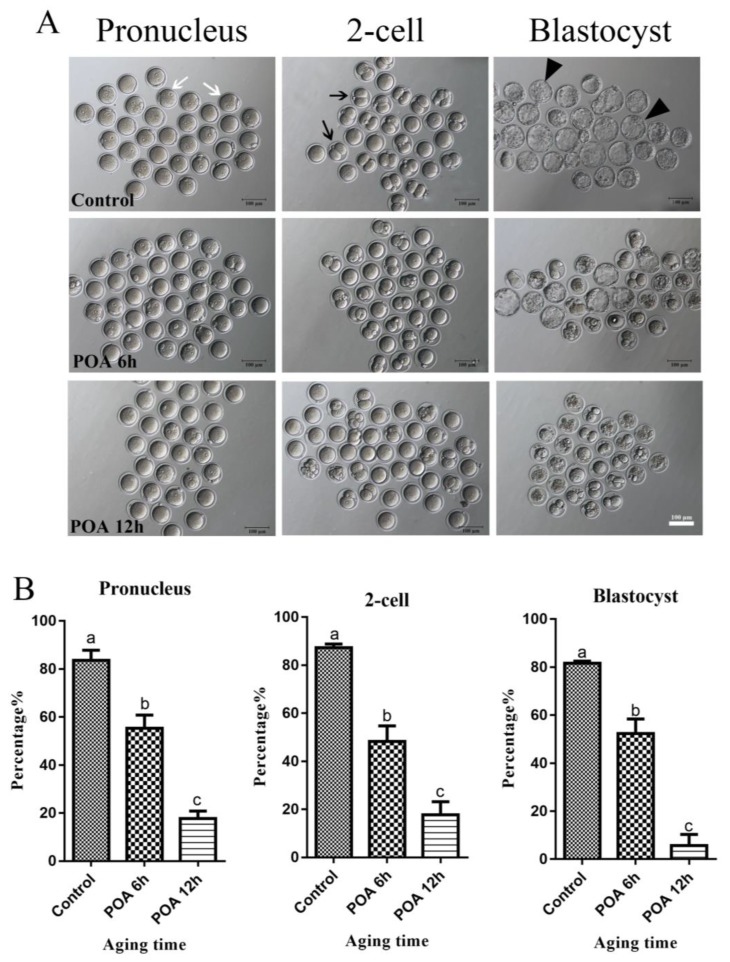
Postovulatory aging (POA) in vitro reduces the rates of fertilization and early embryo development of mouse oocytes. (**A**) Representative images of pronucleus, 2-cell and blastocyst in the control, POA 6 h and POA 12 h groups. The white arrow indicates pronucleus, the black arrow indicates 2-cell and the black triangle indicates blastocyst. Scale bar = 100 μm. (**B**) The rates of pronucleus, 2-cell and blastocyst in the control, POA 6 h, and POA 12 h groups. Data are presented as mean ± SEM from at least three independent experiments. ^a–c^ means not sharing a common superscript are different (*P* < 0.05).

To detect whether POA in vitro impairs the quality of oocytes, we detect the rates of IVF and early embryo development of oocytes aging for 0 (control), 6 and 12 h in vitro. In the control group, most oocytes had a high rates of 2-pronuclei and could develop to 2-cell and blastocyst stages, while with POA time increasing, POA oocytes significantly reduced the rates of 2-pronuclei, 2-cell and blastocyst (n = 164, control vs. n = 104, POA 6 h, vs. n = 119, POA 12 h; 2-pronuclei: 83.7 ± 4.2% vs. 55.3 ± 5.5% vs. 17.7 ± 3.2%; 2-cell: 87.3 ± 1.5% vs. 48.3 ± 6.4% 17.7 ± 3.2%; blastocyst: 81.7 ± 0.9% vs. 52.3 ± 6.1% vs. 5.7 ± 4.7%; *P* < 0.05; Figure 1A,B). These results indicate that POA in vitro have harmful impacts on the mouse oocytes.

### 3.2. Resveratrol Improves the Developmental Potential of POA Oocytes

**Figure 2:**
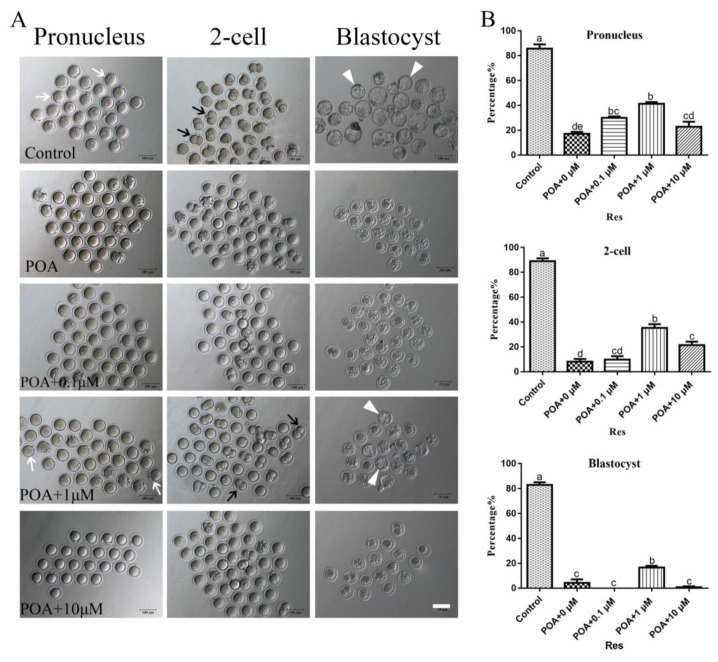
Resveratrol improves the rates of fertilization and early embryo development in POA oocytes. (**A**) Representative images of pronucleus, 2-cell and blastocyst in the control and POA oocytes treated with different concentrations of resveratrol (0, 0.1, 1 and 10 μM) for aging 12 h. The white arrow indicates pronucleus, the black arrow indicates 2-cell and the white triangle indicates blastocyst. Scale bar = 100 μm. (**B**) The rates of pronucleus, 2-cell and blastocyst in the control and POA oocytes treated with different concentrations of resveratrol (0, 0.1, 1 and 10 μM) for aging 12 h. Data are presented as mean ± SEM from at least three independent experiments. ^a–e^ means not sharing a common superscript is different (*P* < 0.05).

The POA 12 h in vitro was chosen to do follow-up research, according to the rates of IVF and blastocyst of oocytes. For testing the effects of resveratrol on POA oocytes, we cultured oocytes for 12 h in vitro with resveratrol at 0, 0.1, 1, and 10 µM and then performed further experiments. According to the results, resveratrol at 1.0 µM significantly increased the rates of fertilization, 2-cell and blastocyst compared with the POA group with 0 µM resveratrol (n = 166, control vs. n = 100, POA + 0 µM Res vs. n = 97, POA + 0 µM Res vs. n = 107, POA + 0.1 µM Res vs. n = 97, POA + 10 µM Res; 2-pronuclei: 85.7 ± 3.5% vs. 17 ± 1.7% vs. 30 ± 1.2% vs. 41.3 ± 1.3% vs. 22.7 ± 4.2%; 2-cell: 89 ± 2.3% vs. 8 ± 2.3% vs. 9.7 ± 2.9% vs. 35.3 ± 3.0% vs. 21.3 ± 2.8%; blastocyst: 83 ± 2.1% vs. 4.3 ± 2.8% 0 ± 0% vs. 16.7 ± 1.3% vs. 0.8 ± 0.8%; *P* < 0.05; Figure 2A,B). The results in 1.0 µM group are better than those in 0.1 or 10 µM resveratrol groups. All these results demonstrate that appropriate resveratrol can improve the ability of fertilization and early embryo development. Therefore, the optimal concentration of resveratrol at 1.0 µM is chosen to perform subsequent research.

### 3.3. Resveratrol Improves the Sperm Binding Ability and Restores the Juno Expression of POA Oocytes

**Figure 3:**
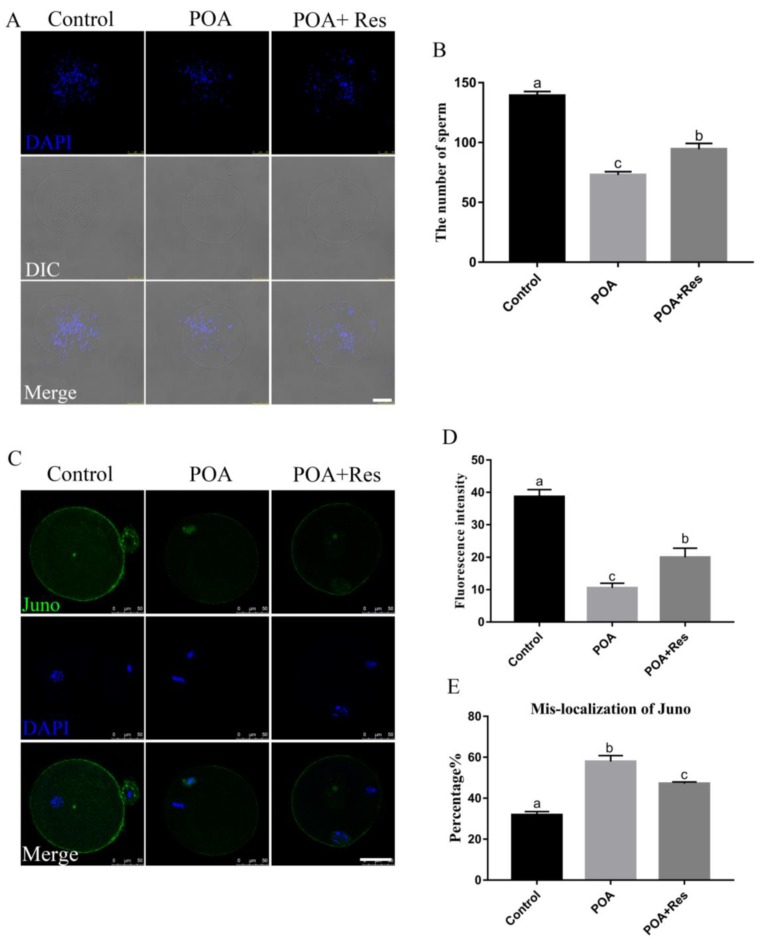
Resveratrol improves the sperm binding ability and restores the Juno expression in POA oocytes. (**A**) Representative images of sperms bound to oocytes in the control, POA and resveratrol-treated POA (POA + Res) groups that were aged for 12 h. Scale bar = 100 μm. (**B**) The number of sperms bound to zona pellucida from the control, POA and POA + Res groups. (**C**) Representative images of Juno in the control, POA and POA + Res groups. Scale bar = 50 μm. (**D**) The fluorescence intensities of Juno in the control, POA and POA + Res groups. (**E**) The rates of mis-localization Juno in the control, POA and POA + Res groups. Data are presented as mean ± SEM from at least three independent experiments. ^a–c^ means not sharing a common superscript is different (*P* < 0.05).

The ability of sperm binding to zona pellucida is an important index to assess the fertilization potential of oocytes. In the POA group, the number of sperms bound to zona pellucida significantly decreased compared with that in the control. After treated with 1 µM resveratrol, the number of bound sperms significantly increased compared to that in the POA oocytes, although not same to the level of control (Figure 3A). The numbers of bound sperms were 139.2 ± 3.5%, n = 78 control vs. 72.9 ± 2.9%, n = 84 POA group, vs. 94.5 ± 4.9%, n = 84 POA + Res group, *P* < 0.05, (Figure 3B). These bound sperms will penetrate zona pellucida to fuse with the oocyte membrane by Juno’s interaction with Izumo, which is as sperm surface [25,30]. According to this method reported, we next detected the expression and localization of Juno on the oocyte membrane. In the control, Juno was located in the oocyte membrane evenly, but it was partially or totally absent in the POA group. After treatment with resveratrol, Juno signal was partially restored compared with that in the POA group (Figure 3C). The fluorescent intensity is 38.7 ± 1.3%, n = 90, control vs. 10.5 ± 0.8%, n = 93, POA vs. 20 ± 1.4%, n = 119, POA + Res; *P* < 0.05, (Figure 3D). The numbers of mis-localized Juno were 31.8 ± 1.6%, n = 116, control vs. 57.9 ± 3.0%, n = 104, POA vs. 43.4 ± 3.0%, n = 114, POA + Res; *P* < 0.05, (Figure 3E). All these data indicate that appropriate resveratrol can improve the sperm binding ability and restore the Juno expression of POA oocytes in vitro.

### 3.4. Resveratrol Maintains Correct Spindle Assembly and Morphology in POA Oocytes

**Figure 4:**
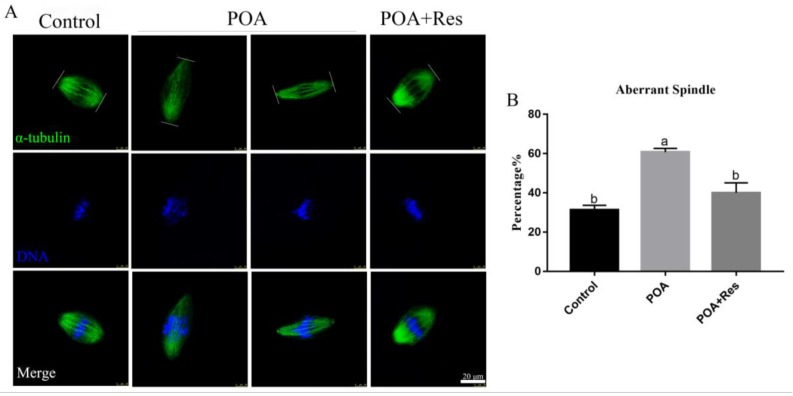
Resveratrol maintains correct spindle assembly and morphology in POA oocytes. (**A**) Representative images of spindle assembly in the control, POA and resveratrol-treated POA (POA + Res) oocytes. Images of spindle (green) and DNA (blue) were observed by the confocal microscopy. Scale bar = 20 μm. (**B**) The percentages of the aberrant spindles in the control, POA, and POA + Res groups. Data are presented as mean ± SEM from at least three independent experiments. ^a,b^ means not sharing a common superscript is different (*P* < 0.05).

If spindle assembly is inaccurate, it will affect the normal process of meiosis and lead to the formation of aneuploidy. Therefore, we detected the effects of POA on spindle assembly and morphology of MII oocytes. In the control group, most spindles were normal morphology. By contrast, spindles displayed a majority of elongated shape in the POA group (Figure 4A). As expected, if POA oocytes were treated with resveratrol, the rate of elongated spindles significantly decreased compared with that of the POA oocytes, similar to the control level. The rates of elongated spindles were 31.2 ± 2.4%, n = 111, control vs. 60.8 ± 1.8%, n = 92, POA vs. 40 ± 5.1%, n = 92, POA + Res; *P* < 0.05, (Figure 4B). These observations reveal that resveratrol can protect spindle morphology from POA in oocytes.

### 3.5. Resveratrol Reduces the Clustered Mitochondrion Distribution in POA Oocytes

**Figure 5:**
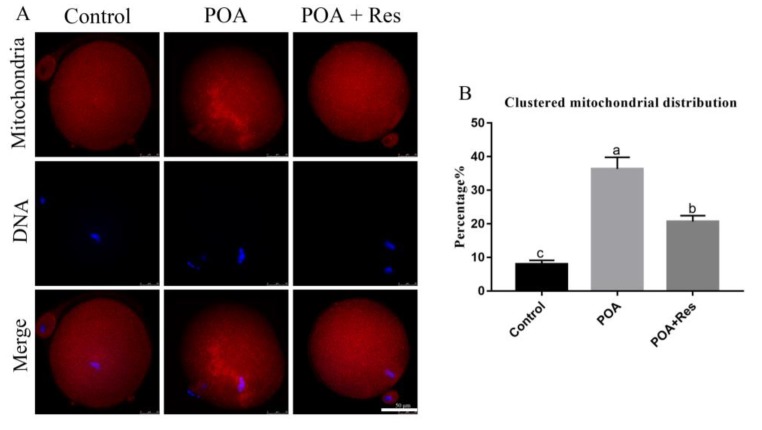
Resveratrol reduces the clustered mitochondrion distribution in POA oocytes. (**A**) Representative images of the mitochondrion distribution in the control, POA and resveratrol-treated POA (POA + Res) oocytes. Clustered distribution is in the POA and homologous distribution is in the control and POA + Res groups. Scale bar = 50 μm. (**B**) The rates of clustered mitochondrial distribution in the control, POA and POA + Res groups. Data are presented as mean ± SEM from at least three independent experiments. ^a–c^ means not sharing a common superscript is different (*P* < 0.05).

Dysfunctional mitochondria can lead to the failure of the process of fertilization and early embryo development. In order to evaluate the effects of POA on MII oocyte mitochondria, MitoTracker Deep Red was used to detect the mitochondrion distribution [29]. Most control oocytes showed a typical homogenous distribution, while POA oocytes showed clustered distribution of mitochondria (Figure 5A). When oocytes were aged for 12 h with resveratrol, the rate of clustered distribution significantly decreased compared to that in the POA oocytes, but not similar to the control. The percentages of clustered mitochondrion distribution were 8 ± 1.2%, n = 85, control vs. 36.3 ± 3.5%, n = 107, POA vs. 20.7 ± 1.8%, n = 107, POA + Res; *P* < 0.05, (Figure 5B). Thus, resveratrol can partially reduce the clustered mitochondrion distribution in POA oocytes.

### 3.6. Resveratrol Decreases the Intracellular ROS Accumulation in POA Oocytes

**Figure 6:**
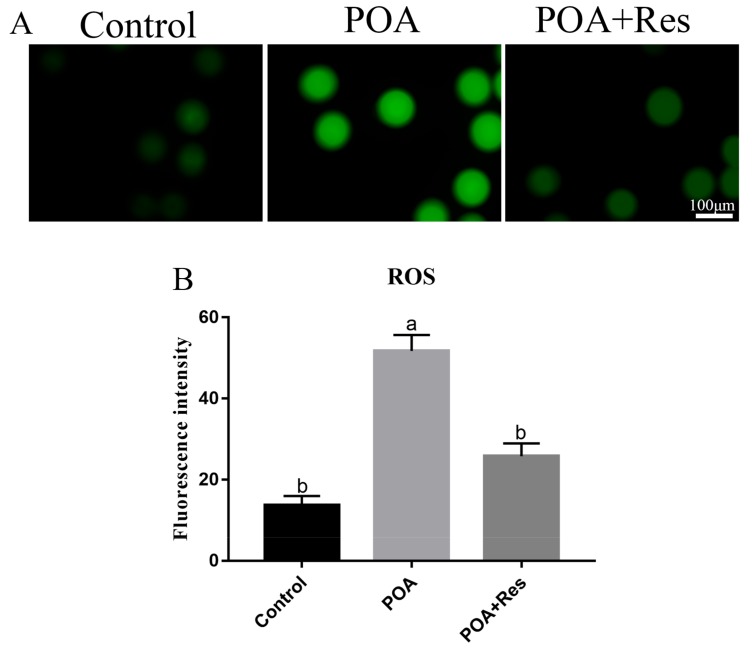
Resveratrol decreases the intracellular ROS accumulation in POA oocytes. (**A**) Representative images of reactive oxygen species (ROS) (green) in the control, POA and resveratrol-treated POA (POA + Res) oocytes detected with Reactive Oxygen Species Assay Kit. Scale bar = 100 μm. (**B**) The fluorescence intensities of ROS in the control, POA and POA + Res groups. Data are presented as mean ± SEM from at least three independent experiments. ^a,b^ means not sharing a common superscript is different (*P* < 0.05).

The production of intracellular ROS is connected with mitochondrial functions, so next we tested the ROS level of MII oocytes with Reactive Oxygen Species Assay Kit by fluorescent analysis. In POA oocytes, the fluorescent signal was higher than that in the control, while if POA oocytes were treated with resveratrol, the fluorescent signal significantly decreased compared with that in the POA oocytes (Figure 6A). The numbers of fluorescence intensity are 13.6 ± 2.3%, n = 101, control vs. 51.7 ± 3.9%, n = 97, POA vs. 25.8 ± 3.1%, n = 89, POA + Res; *P* < 0.05, and the control group shows no significant difference to the POA + Res group (Figure 6B). All these data demonstrate that resveratrol can reduce the accumulation of intracellular ROS in POA oocytes.

### 3.7. Resveratrol Alleviates the Early Apoptosis of POA Oocytes

**Figure 7:**
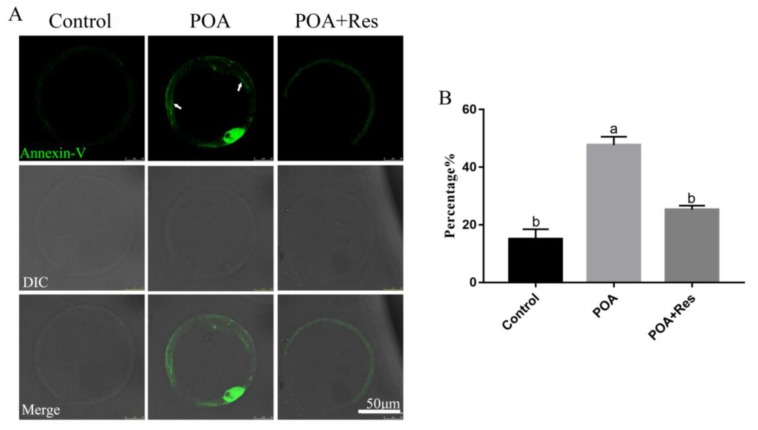
Resveratrol alleviates the apoptosis of POA oocytes. (**A**) Representative images of apoptosis (green) in the control, POA and resveratrol-treated POA (POA + Res) oocytes. The fluorescent signals only at membrane (white arrow) are regarded as early apoptosis. Scale bar = 50 μm. (**B**) The rates of early apoptosis in the control, POA and POA + Res groups. Data are presented as mean ± SEM from at least three independent experiments. ^a,b^ means not sharing a common superscript is different; *P* < 0.05.

The intracellular ROS accumulation beyond one threshold will impair cell, thus leading to apoptosis. Next we examined the early apoptosis by detecting Annexin V signals. In the MII POA oocytes, the fluorescent signal on the membrane was higher than that in the control; while in the resveratrol-treated POA oocytes, the fluorescent signal on the membrane was significantly decreased compared to that in the POA oocytes (Figure 7A). The percentages of early apoptosis are 15±3.5%, n = 102, control vs. 47.7 ± 2.9%, n = 123, POA vs. 25.3 ± 1.4%, n = 163, POA + Res; *P* < 0.05, and the control group shows no significant difference to the POA + Res group (Figure 7B). In conclusion, resveratrol can alleviate the early apoptosis of POA oocytes.

### 3.8. Resveratrol Restores the Abnormal Level of H3K9me2 in POA Oocytes

**Figure 8:**
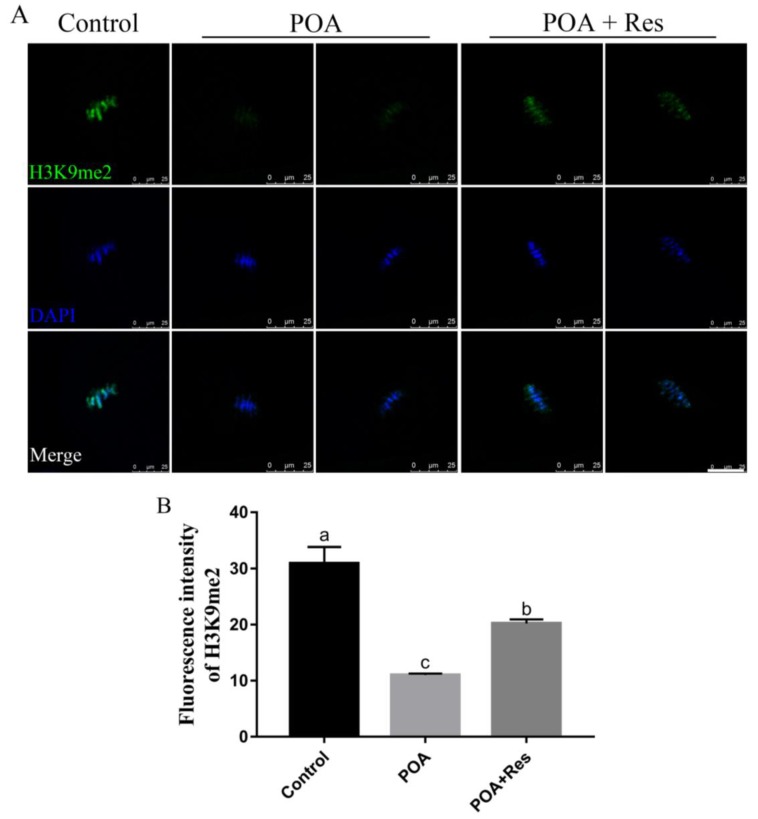
Protective effects of resveratrol on the level of H3K9me2 in POA oocytes. (**A**) Representative images of H3H9me2 (green) in the control, POA and resveratrol-treated POA (POA + Res) oocytes. Scale bar = 25 μm. (**B**) The fluorescence intensities of H3H9me2 in the control, POA and POA + Res groups. Data are presented as mean ± SEM from at least three independent experiments. ^a–c^ means not sharing a common superscript is different; *P* < 0.05.

Next we examined the histone lysine methylation of H3K9me2 related to epigenetic modification in MII oocytes. In the POA oocytes, the fluorescent signal of H3K9me2 decreased significantly compared to that in the control; while in the resveratrol-treated POA oocytes, the fluorescent signal increased significantly compared to that in the POA oocytes (Figure 8A). The numbers of fluorescence intensity are 30.91 ± 2.9%, n = 102, control vs. 11.02 ± 0.3%, n = 83, POA vs. 20.23 ± 0.7%, n = 111, POA + Res; *P* < 0.05 (Figure 8B). These data indicate that resveratrol can restore the abnormal histone lysine methylations of POA oocytes.

## 4. Discussion

In the whole world, 1% of all babies were born with the help of ART [31]. In some countries the rate of birthing through ART is even higher, such as in Australia 2.7% (1/37) of all babies (Australian Bureau of Statistics. ‘Births, 3301.0, 2007). During ART process, the oocyte POA is inevitable and the adverse effects of POA should be avoided. In this study, we investigated the effects of resveratrol on POA oocytes and the mechanisms through evaluating oocytes quality, sperm binding, cytoskeletal integrality, mitochondria and reactive oxidative stress, and early apoptosis. The results show that the appropriate resveratrol can significantly improve the quality of POA oocytes in vitro and enhance the rates of fertilization and blastocyst, which will be very helpful in ART.

ART is not only used in the infertility of old women, but also used in young women for various reasons, such as surrogacy, tubal obstruction, poor sperm quality, and to conserve oocytes before chemotherapeutic drugs for cancer and polycystic ovarian syndrome (PCOS). Any oocyte undergoing POA will exhibit a reduction in quality. Resveratrol is well recognized as an antioxidant and anti-aging substance, so we hypothesized that utilizing resveratrol can improve the quality of POA oocytes in vitro. The rates of fertilization and blastocyst are the most important and direct indicator of high-quality oocytes. Blastocyst is always used as the index for the early embryo development and transplanted into the womb of female who gives birth. The sperm binding to the zona pellucida is the first step for fertilization, since oocytes are surrounded by zona pellucida composed of ZP1, ZP2, and ZP3. Capacitated sperm binds to the N-terminal domain of ZP2 via the sperm protein IZUMO1 and its interaction with the oocyte membrane receptor Juno to promote gamete fusion during fertilization [32,33]. After fertilization, Juno will be lost from the plasma membrane of oocyte, accompanied with ZP2 modification. Therefore, the extra sperms cannot bind to the zona pellucida. POA leads to the premature lost of Juno from oocyte, which will impair sperm binding and fertilization. Resveratrol can reduce the loss of sperm binding sites by stabilizing Juno in POA oocytes so to increase the fertilization potential. More importantly, the defects caused by POA can be restored partially or completely by adding appropriate resveratrol during the aging process in vitro.

High quality oocytes undergo complete cytoplasmic maturation, which mainly includes cytoskeletal integrality, organelle reorganization, and other factors playing roles in fertilization and early embryo development [34]. Aberrant cytoskeleton is considered an important element for increasing fetal miscarriage and incidence of infertility. Microtubules are the most important ingredient of the cytoskeleton since the meiotic spindle is made up from microtubules which enable faithful chromosome segregation in the oocyte. POA increases the aberrant spindle. Resveratrol can maintain the correct spindle assembly in POA oocytes which indicates that resveratrol improves the cytoplasmic maturation of the oocyte by protecting the cytoskeletal integrality.

The second issue of cytoplasmic maturation is organelle reorganization. The mitochondria are important organelles, connected to energy supply, ROS, and apoptosis. During maturation of mouse oocytes, mitochondria will move from the perinuclear area to the cytoplasm, namely homologous distribution to supply energy for fertilization and early embryo development. Failed cytoplasmic maturation is connected with abnormal mitochondrion migration [35,36]. Resveratrol can preserve homologous mitochondrion distribution in POA oocytes. Therefore, resveratrol can maintain successful mitochondrion migration from the perinuclear area to the cytoplasm in POA oocytes.

Oxidative stress acts as the initiator for a cascade of events that make the aged oocyte phenotype, such as impairing lipids, proteins, and DNA [37]. ROS accumulation is significantly decreased in resveratrol-treated POA oocytes compared with POA oocytes, suggesting that resveratrol plays the part roles of antioxidant effect on POA oocytes. Accumulation of intracellular ROS has been demonstrated to result apoptosis [37,38]. The ending of POA oocytes is cell death by initiating apoptosis pathways if not repaired. The percentage of early apoptosis is significantly decreased in the resveratrol-treated POA oocytes, indicating resveratrol acts an anti-apoptotic effect to in the POA oocytes. All these combined data related to mitochondrion demonstrate that resveratrol improves cytoplasmic maturation from organelle reorganization perspective.

Epigenetic modifications are susceptible to aging. Previous studies have shown that histones were methylated in the germinal vehicle (GV) oocytes, such as H3K9me2, and methylation was kept in MII oocytes [39,40]. H3K9me2 is widely present in the genome and plays an important role in cell differentiation [41,42]. Additionally, H3K9me2 has valuable functions in protecting the genome in mouse eggs as well as in preimplantation embryos [43]. If the modification is abnormal during this process, the embryos will develop abnormally and even die before implantation. The levels of histone methylation were decreased in the POA oocytes, proved by the decreased level of H3K9me2. As expected, the levels of histone methylation were increased in the resveratrol-treated POA oocytes compared to those of POA oocytes. Thus, resveratrol can protect the loss of methylation of H3K9me2 and protect the epigenetic modification of POA oocytes to improve the subsequent development of blastocyst.

## 5. Conclusions

In conclusion, resveratrol can improve the quality of POA oocytes in vitro to enhance the rates of fertilization and blastocyst, by stabilizing Juno for fertilization and protecting the cytoplasmic maturation including cytoskeletal integrality, organelle reorganization, and epigenetic modifications. These results will be very helpful in ART.
